# Secretary’s Report Georgia State Commission on Tuberculosis

**Published:** 1905-05

**Authors:** 


### SECRETARY'S REPORT GEORGIA STATE COMMISSION ON TUBERCULOSIS.

Your Secretary begs leave to present the following preliminary report: The Georgia State Commission on Tuberculosis was created for the specific purpose of ascertaining the status of tuberculosis in the State and of recommending the adoption on the part of the State of such means and measures as would tend to limit the spread and to suppress the disease. As a result of the organization meeting held in Macon, October 19, 1904, the Commission came to realize that the task set before it was a large one, and that the difficulties and obstacles in the way of its successful fulfilment were many and varied. The Commission was hampered at the outset by entire lack of pecuniary resource other than that voluntarily subscribed by the individual members in pursuance of a resolution introduced at the Macon meeting. This assessment, nearly all of which was promptly paid, amounted to $95.00, and was to be devoted to the necessary expenses incurred by the Secretary in the general work of the Commission. It was with this small and totally inadequate sum that the task of gathering statistics throughout the eleven Congressional districts in the State was undertaken.

The drafting of some specific form for distribution to the physicians in the State to secure from them the desired statistical data was next considered, and after consultation with the Chairman, the form appended was adopted. There has been some criticism offered concerning the comprehensiveness and usefulness of the form, but as the duty of the Commission was prescribed by the Act creating it, it was deemed expedient to adhere to the letter of the resolution, and in consequence the inquiry is designed only to cover the five propositions enumerated in the Bill, all other interrogatories having been deleted therefrom. To make this point clear, the Act is herewith inserted.

*Resolved by the Senate, the House concurring, That the Governor of the State of Georgia be and he is hereby authorized to raise a medical commission to consist of one physician from each Congressional district and ten from the State at large, who shall make a thorough investigation touching the propositions hereinafter set forth and numbered, and embody the same in a report to be submitted to the next session of the General Assembly:* (1) *The number of cases of tuberculosis in the State of Georgia.* (2) *The annual number of deaths from tuberculosis in the State of Georgia.* (3) *The number of cases contracted in the State and the number imported.* (4) *The measures that have been taken in the various localities for preventing the spread of the disease.* (5) *Whether or not the physicians of the State are in favor of taking measures for its prevention.*

*Be it further resolved, That the Commission shall serve without compensation and without entailing any expense whatever upon the State of Georgia in connection with the said investigation.*

The work of gathering statistics was proceeded with as follows: The number of physicians, without reference to school affiliation in the eleven Congressional districts, was roughly estimated, and a sufficient number of blanks was furnished each district member of the Commission to cover his allotted territory. The various agencies by means of which the inquiry could be furthered were utilized, and each member of the Commission from the Congressional districts was requested to make a roster, compiling it from any and all authentic sources of information of the physicians residing in his district. This task was aided by official maps which were presented by the Assistant Commissioner of Agriculture, the Hon. R. A. Wright, to whom the appreciative thanks of the Commission are hereby tendered. It was suggested that the blanks be distributed by mailing them with a stamped and addressed envelope enclosed for reply. As the expense of this method of distribution had to be borne by the sender individually, it was not made mandatory, but was offered as the best means of securing prompt replies. The plan was adopted by the majority of the district commissioners, and it is entirely likely that the modicum of success attained in collecting the data was due to it. The members of the Commission from the State at large were requested to cooperate with the district members in their work when called upon to do so. This they cheerfully consented to do, and in some instances have rendered most valuable aid.

In January, 1905, at the suggestion of the Chairman, the Secretary wrote a circular letter to the district members asking for a preliminary report in order to form some conception of the progress of the work, and to indicate its further course. The response was not as general as might have been desired, but was sufficient to show from the tentative reports that the returns were utterly disproportionate to the number of inquiries sent out. This was not attributed to indifference on the part of the profession generally toward the objects of the Commission, but rather to some other cause, procrastination or difficulty in sifting from inaccurate or unmethodically-kept records or supplying from memory cases about which information was desired.

At this time it became apparent that the scope of the Commission should be appropriately limited to the task of collecting statistics foregoing any enlargement of function. The slow and tedious process of gathering information by a canvas of physicians gave every promise of consuming most of the time allotted to the Commission, and the discouraging meagerness of response from these, the only dependable and available sources of information, led to the conviction that the function of the Commission for the present should reside solely in the acquisition of facts touching the numerical position of tuberculosis in the State. The promulgation to the public of instructive literature on the subject of the disease through the usual channels of diffusion was abandoned, at least for the time, or relegated to another body of somewhat similar intent. The preliminary reports were retained by the Secretary, and received such additions and emendations as were sent in until the time approached for this meeting, when they were all collected and epitomized, as will appear from the table. The Secretary desires to express sincere thanks to Dr. Alex. Mack for his valued assistance in tabulating the reports:

